# Predictive value of ^18^F-FDG PET/CT radiomics for EGFR mutation status in non-small cell lung cancer: a systematic review and meta-analysis

**DOI:** 10.3389/fonc.2024.1281572

**Published:** 2024-02-01

**Authors:** Ning Ma, Weihua Yang, Qiannan Wang, Caozhe Cui, Yiyi Hu, Zhifang Wu

**Affiliations:** ^1^ Department of Nuclear Medicine, First Hospital of Shanxi Medical University, Taiyuan, China; ^2^ Molecular Imaging Precision Medical Collaborative Innovation Center, Shanxi Medical University, Taiyuan, China

**Keywords:** non-small cell lung cancer, EGFR mutation, ^18^F-FDG PET/CT, meta-analysis, radiomics

## Abstract

**Objective:**

This study aimed to evaluate the value of ^18^F-FDG PET/CT radiomics in predicting EGFR gene mutations in non-small cell lung cancer by meta-analysis.

**Methods:**

The PubMed, Embase, Cochrane Library, Web of Science, and CNKI databases were searched from the earliest available date to June 30, 2023. The meta-analysis was performed using the Stata 15.0 software. The methodological quality and risk of bias of included studies were assessed using the Quality Assessment of Diagnostic Accuracy Studies 2 and Radiomics Quality Score criteria. The possible causes of heterogeneity were analyzed by meta-regression.

**Results:**

A total of 17 studies involving 3763 non-small cell lung cancer patients were finally included. We analyzed 17 training cohorts and 10 validation cohorts independently. Within the training cohort, the application of ^18^F-FDG PET/CT radiomics in predicting EGFR mutations in NSCLC demonstrated a sensitivity of 0.76 (95% CI: 0.70-0.81) and a specificity of 0.78 (95% CI: 0.74-0.82), accompanied by a positive likelihood ratio of 3.5 (95% CI:3.0-4.2), a negative likelihood ratio of 0.31 (95% CI: 0.24-0.39), a diagnostic odds ratio of 11.0 (95% CI: 8.0-16.0), and an area under the curve (AUC) of 0.84 (95% CI: 0.80-0.87). In the validation cohort, the values included a sensitivity of 0.76 (95% CI: 0.67-0.83), a specificity of 0.75 (95% CI: 0.68-0.80), a positive likelihood ratio of 3.0 (95% CI:2.4-3.8), a negative likelihood ratio of 0.32 (95% CI: 0.24-0.44), a diagnostic odds ratio of 9 (95% CI: 6-15), and an AUC of 0.82 (95% CI: 0.78-0.85). The average Radiomics Quality Score (RQS) across studies was 10.47 ± 4.72. Meta-regression analysis identifies the application of deep learning and regions as sources of heterogeneity.

**Conclusion:**

^18^F-FDG PET/CT radiomics may be useful in predicting mutation status of the EGFR gene in non-small cell lung cancer.

**Systematic review registration:**

https://www.crd.york.ac.uk/PROSPERO, identifier CRD42022385364.

## 1 Introduction

Lung cancer is one of the most prevalent malignant tumors with high incidence and mortality rates (1, 2). Non-small-cell lung cancer (NSCLC) accounts for approximately 85% of primary lung cancers, and most patients are diagnosed with an advanced stage of the disease, leading to a 5-year survival rate of less than 20%. The use of tyrosine kinase inhibitors targeting the epidermal growth factor receptor (EGFR) has been an effective treatment of improving the prognosis of NSCLC patients with EGFR mutation (3, 4). Therefore, the early identification of EGFR gene mutation status in NSCLC patients is critical. Nevertheless, gene detection methods usually require an invasive tissue or cell biopsy, a process that is time-consuming and possibly risky. Therefore, it is essential to develop a non-invasive and faster detection method to predict EGFR mutation status.^18^F-fluorodeoxyglucose positron emission tomography/computed tomography (^18^F-FDG PET/CT) is routinely used for tumor staging, treatment decision-making, and response monitoring in NSCLC patients (5, 6). Previous studies have shown that semi-quantitative parameters derived from ^18^F-FDG PET/CT, including maximum standard uptake value (SUVmax) and total lesion glycolysis (TLG), have reasonable diagnostic utility in detecting EGFR mutation status in NSCLC patients. However, meta-analysis indicates that SUVmax has a low pooled sensitivity and specificity in predicting EGFR mutation status in NSCLC patients (7, 8). Therefore, it is necessary to investigate additional parameters such as radiomic features of ^18^F-FDG PET/CT to predict EGFR mutation status in NSCLC.

Radiomics is an emerging field that analyzes quantitative medical images to extract a large number of objective and quantitative image features that are correlated with clinical, pathological, molecular, and genetic features that may reflect tumor genetic phenotypes by utilizing artificial intelligence algorithms (9). Previously published studies have exhibited significant variations in methodology and outcomes (10, 11). Thus, Consequently, the objective of this study is to conduct a meta-analysis of published studies on ^18^F-FDG radiomics for predicting EGFR mutation status in patients with NSCLC.

## 2 Materials and methods

This study followed the Preferred Reporting Item of the Guidelines for Systematic Reviews and Meta-Analysis (PRISMA) and was registered in PROSPER (CRD42022385364, https://www.crd.york.ac.uk/prospero).

### 2.1 Literature search

The PubMed, Embase, Web of Science, Cochrane Library, and CNKI databases were searched by two independent observers to identify eligible studies up to June 30, 2023. The searches used a combination of subject headings and free terms, including “radiomics”, “texture analysis”, “artificial intelligence”, “lung cancer”, “PET/CT”, “EGFR”, and “^18^F-FDG”. The search strategies are shown in **Supplementary Tables S1**-**S5**.

### 2.2 Inclusion and exclusion criteria

We selected publications for review if they met several of the following inclusion criteria: (1) All patients were NSCLC patients with pathology confirmation and underwent ^18^F-FDG PET/CT scans. (2) Radiomics or deep learning algorithms applied to predict EGFR mutation status. (3) The number of true positives (TP), false positives (FP), true negatives (TN), and false negatives (FN) must be reported or quantified in the studies. Exclusion criteria: (1) Reviews, meta-analyses, conference abstracts, editorials, and notes. (2) Duplicated and irrelevant studies. (3) Studies where diagnostic data could not be obtained.

### 2.3 Data extraction

Data from each eligible study were independently extracted by two reviewers. The following data were collected: first author, publication year, country/region, study object, study design, blindness to EGFR mutation results when reviewing the PET/CT image, sample size, EGFR mutation rate, patient characteristics (age, gender, and ^18^F-FDG injection dose), tumor segmentation, feature extraction, feature type, validation and radiomics algorithms, receiver operating characteristic curve (AUC), sensitivity, specificity, TP, FP, TN, and FN. In situations where a study reported accuracy data for multiple models, we utilized the 2 × 2 tables for the model with the highest AUC.

### 2.4 Quality assessment

Two independent investigators used the Quality Assessment for Diagnostic Accuracy Studies-2 (QUADAS-2) and the Radiomics Quality Score (RQS) to assess the methodological quality and risk of bias of the included studies (12, 13). Where there were differences of opinion, we had recourse to a third reviewer for conflict resolution. QUADAS-2 primarily includes assessment of risk of bias and clinical applicability. Risk of bias includes “patient selection”, “index test”, “reference standard”, and “flow and timing”, while clinical applicability requires assessment of the first three. The quality of radiomics processes and reports was evaluated using the Radiomics Quality Score (RQS), which comprised 16 dimensions. The total scores ranged between -8 and +36. A score between -8 and 0 was equivalent to 0%, while a score of 36 was equivalent to 100%. The inter-reviewer agreement for each item of the RQS was quantified using the modified Fleiss kappa statistic, tailored for ordered variables. Overall inter-reviewer agreement, including for the RQS, was assessed using intergroup correlation coefficients (ICCs), calculated via a single-source, two-way random effects model to ascertain absolute agreement between reviewers.

### 2.5 Statistical analysis

Meta-analysis was performed by utilizing Stata 15.0 software (Stata Corp, College Station, Texas, USA) and the MIDAS bivariate random-effects model. Heterogeneity among the studies included in our analysis was assessed using the Cochran *Q* test and the *I^2^* statistic. The significance level was set at *P*<0.05. According to the Cochrane Handbook for Systematic Reviews of Diagnostic Test Accuracy, the *I^2^* value reflects the degree of heterogeneity, with an *I^2^* value of more than 50% indicating high heterogeneity (14). The effectiveness of ^18^F-FDG PET/CT in detecting EGFR mutation status in NSCLC patients was evaluated by calculating the pooled statistics of sensitivity (SEN), specificity (SPE), positive likelihood ratio (PLR), negative likelihood ratio (NLR), diagnostic odds ratio (DOR), and their corresponding 95% confidence intervals (CI). Predictive accuracy was also evaluated using summary receiver operating characteristic and area under the curves. Spearman’s correlation coefficient was calculated using Meta-Disc 1.4 software (Ramon y Cajal Hospital, Madrid, Spain) to investigate the potential threshold effect. A threshold effect was considered present if *r* > 0.5 and *P* < 0.05. Publication bias was assessed using the Deeks funnel plot, where significant asymmetry was indicated if *P <*0.10. Sensitivity analysis was to observe the stability of the synthetic results. In our meta-regression, aimed at pinpointing sources of heterogeneity, analyzed covariates such as blinding to EGFR mutation results in PET/CT reviews (yes or unclear), modeling methods (deep learning or radiomics algorithms), sample (<130 or ≥130), study focus (NSCLC or ADC only), and the radiomics software (Pyradiomics or others), publication year (before or after 2022). Factors in model construction included the integration of clinical information,gender number (≥50 or <50), number of smokers (≥100 or <100), Radiomics Quality Score (RQS ≥12 or <12), and region (mainland China or others). For assessing clinical utility, we calculated posttest probabilities and created Fagan plots.

## 3 Results

### 3.1 Literature search

**Figure 1:**
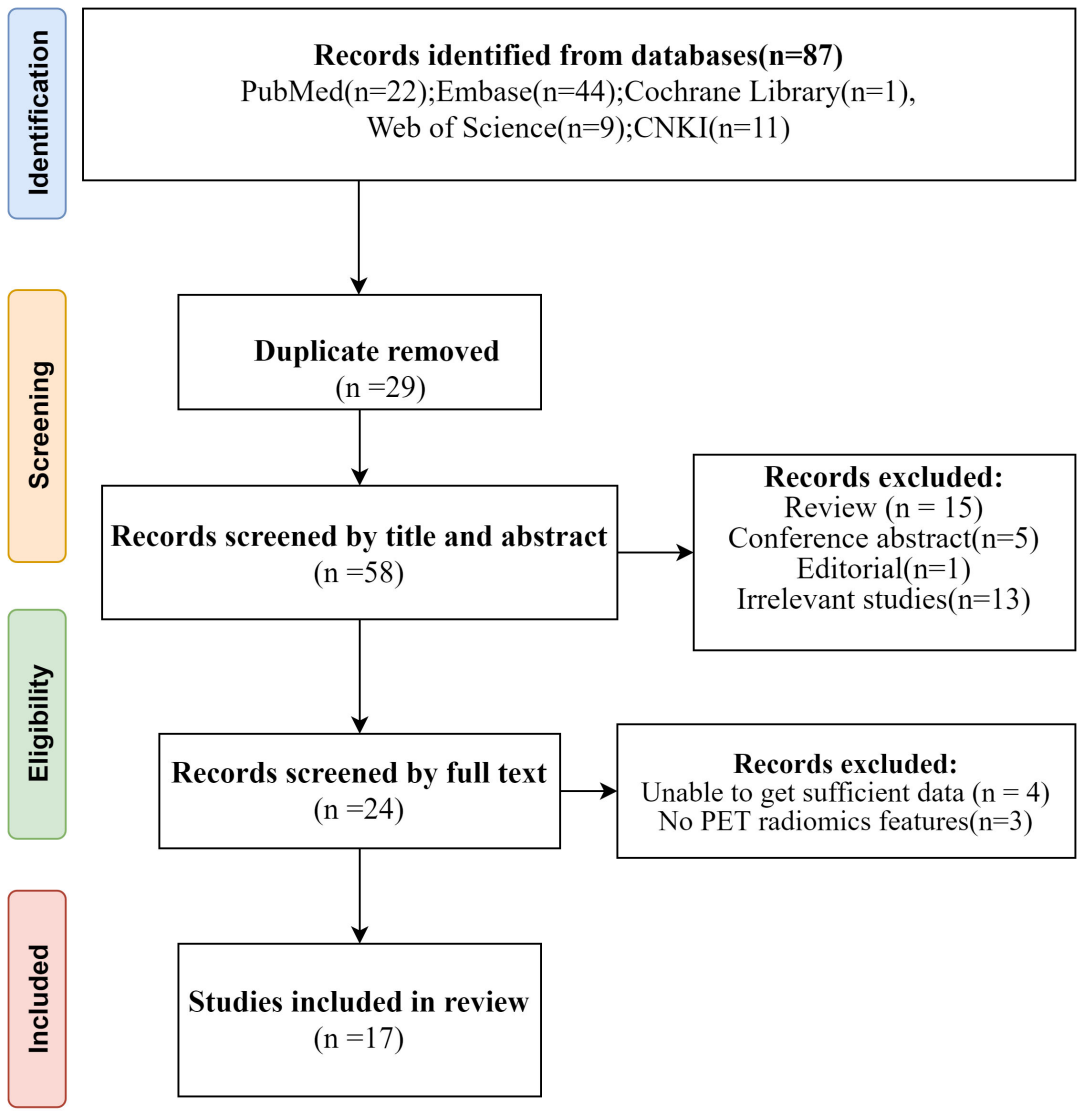
Flow diagram of study selection.

A comprehensive search was conducted through various databases including PubMed, Embase, Cochrane Library, Web of Science, and CNKI. Initially, a total of 87 studies were found. After removing 29 duplicate articles, the remaining articles were screened by two independent reviewers based on their titles and abstracts. 5 conference abstracts, 15 reviews, 1 editorial, 13 irrelevant studies and 1 note were excluded subsequently. Further assessment of the full texts led to the exclusion of 4 studies with insufficient data and 3 studies without PET radiomics feature. Ultimately, 17 diagnostic studies that met the inclusion criteria were included in the analysis. The PRISMA flow-chart of the literature search of our systematic review and meta-analysis is presented in **Figure 1**. All studies included were retrospective cohort studies.

A total of 3763 patients were included, and the sample sizes of the studies ranged from 50-583, Our study’s training cohort comprised 2877 individuals, averaging 169 participants per study and a median size of 127, while the validation group encompassed 1021 individuals. In the training set, 1239 patients reported a smoking history, and 1321 were female. Additionally, nine of the studies integrated clinical characteristics, including demographic data (age, gender), smoking history, and tumor stage, to enhance EGFR mutation prediction accuracy. Our analysis encompassed 17 studies: 15 from China, one from Canada, and one utilizing public datasets (‘TCGA LUAD’ and ‘NSCLC Radiogenomics’). Of these, seven specifically investigated lung adenocarcinoma (ADC), while the others included ADC and other NSCLC subtypes. The radiomic features analyzed were diverse, spanning first-order, texture, shape, size, and deep learning features. Commonly employed feature types included Gray Level Co-occurrence Matrix, Gray Level Dependence Matrix, Gray Level Run Length Matrix, Gray Level Size Zone Matrix, and Neighborhood Gray Tone Difference Matrix. In radiomics analysis, five studies utilized Pyradiomics for feature extraction. Of the studies reviewed, thirteen used classical machine learning algorithms and four used deep learning approaches. The literature’s publication dates range from 2019 to 2023. The literature’s publication dates range from 2019 to 2023. The specific characteristics of the included literature are shown in **Tables 1**, **2**.

**Table 1:** Characteristics of the included studies.

Study ID	Study object	Sample size	Age	Gender (M/F)	Country	EGFR mutation rate	^18^F-FDG injection dose	Segmentation Method (Software/Algorithm)	Feature Extraction	Features Type	Modeling method	Validation
Chang et al. (15) (2021)	ADC	583	EGFR (–):63 (56–67) EGFR(+):61 (53–67)	278/305	China	49.40%	0.10-0.15 mci/kg	Semi-automatic (ITK-SNAP)	Artificial Intelligence Kit	Histogram, GLCM, Formfactor, GLSZM, RLM	logistic regression	Split Sample, 100–folds leave-group-out cross-validation
Ruan et al. (16) (2022)	NSCLC	100	EGFR(-):66 EGFR(+):63	58/42	China	46%	0.10-0.2 mci/kg	Manual (LIEFx)	LIEFx	Intensity, shape, size, and texture features	LLR, SVM	Split sample
Wang et al. (17) (2022)	NSCLC	161	EGFR(-):62.48 ± 10.89 EGFR(+):58.47 ± 11.42	106/55	China	38.51%	4.44-5.55 MBq/kg	Manual (MITK)	Pyradiomics	First-order, second-order and higher-order	GBDT	Split sample
Mu et al. (18) (2020)	NSCLC	616	EGFR (–):63.26 ± 8.94 EGFR(+):62.79 ± 8.65	235/194	China	46.59%	4.38 ± 1.0 MBq/kg	Manual (ITK-SNAP)	Python	Deep learning features	Res-Net	External validation
Zhang et al. (19) (2020)	NSCLC	173	EGFR (–):32~83 EGFR(+):27~86	115/58	China	41.04%	5.55 MBq/KG	Semi-automatic (ITK-SNAP)	Pyradiomics	First-order, second-order and higher-order	RF, SVM, logistic regression	10-fold cross-validation
Liu et al. (20) (2020)	NSCLC	51	EGFR (–):61.48 ± 9.12 EGFR(+):58.21 ± 12.06	28/23	China	52.94%	4.0 MBq/kg	Manual (Medex)	Research Omni Kinetics	Textural features, GLCM, RLM, Shape features	Univariate analysis	NR
Huang et al. (21) (2022)	NSCLC	195	EGFR (–):62.54 ± 11.26 EGFR(+):58.5 ± 10.48	123/72	China	49.23%	5.55 MBq/kg	Semi-automatic (MITK)	Pyradiomics	First-order, second-order and higher-order, deep learning feature	multivariate logistic regression	Split sample
Zhao et al. (22) (2022)	ADC	88	EGFR (–):61.46 ± 11.58 EGFR(+):61.29 ± 11.77	49/39	China	42.05%	3.7-7.4 MBq/kg	Semi-automatic (LIEFx)	LIEFx	Histogram, shape, GLCM, NGLDM,GLRLM,GLZLM	logistic regression	Split sample
Yang et al. (23) (2021)	ADC	114	EGFR (–):60.80 ± 9.8 EGFR(+):61.20 ± 8.4	64/50	China	46.49%	3.7-4.44 MBq/kg	Manual (LIEFx)	LIEFx	Basic, Histogram, shape features, GLCM, GLRLM, NGLDM, GLZLM	logistic regression	10-fold cross-validation
Li et al. (24) (2019)	NSCLC	115	63 (28–77)	53/62	China	44.35%	4 MBq/kg	Semi-automatic (ImageJ 1.50i)	MATLAB	Morphological, grayscale statistic, GLCM	logistic regression	10-fold cross-validation
Zhang et al. (25) (2020)	ADC	248	EGFR (–):63.41 ± 9.71 EGFR(+):62.25 ± 8.58	135/113	China	53.63%	350-550 MBq	Semi-automatic (LIEFx)	LIEFx	Basic features, Histogram, shape, Texture features	multivariate logistic regression	Split sample
Wang et al. (26) (2019)	NSCLC	127	EGFR (–):60 ± 11 EGFR(+):60 ± 9	76/51	China	53.53%	3.70-5.55 MBq/kg	Manual (HuiYiHuiYing)	HuiYiHuiYing	Shape, intensity, texture, wavelet features	logistic regression	NR
Yin et al. (11) (2021)	ADC	301	EGFR (–):63.5 (28–74) EGFR(+):63 (37–75)	162/139	China	50.83%	4.2 MBq/kg	Manual (3D Slicer)	Pytorch, scikit-learn	Deep learning features	SE-ResNet, SVM	Split sample
Nair et al. (27) (2021)	NSCLC	50	NR	32/18	Canada	42%	15 mCi	Manual (OsiriX)	MATLAB	first-order, volume, higher order and texture features	logistic regression	Leave-One-Out Cross-Validation
Li et al. (28) (2022)	ADC	179	EGFR (–):59(53.45-64) EGFR(+):60(53-66.3)	76/103	China	58.66%	0.10–0.15 mCi/kg	Manual (Artificial Intelligence Kit)	Artificial Intelligence Kit	first-order, shape, GLCM, GLDM,GLRLM,GLSZM, NGTDM	logistic regression	Split sample
Chen et al. (29) (2022)	NSCLC, ADC	147	EGFR (–):68.56 ± 9.97 EGFR(+):68.59 ± 9.33	94/53	Public datasets	25.17%	NR	Semi-automatic	PyRadiomics	first-order, shape, GLCM, GLDM GLRLM, GLSZM, NGTDM, Deep learning features	ResNet,SVM	Nested five-folds cross validation
Gao et al. (30) (2023)	ADC	515	EGFR (–):64.83 (9.11) EGFR(+):63.65 (9.22)	251/264	China	60.77%	3.70-5.55 MBq/kg	Semi-automatic (3D Slicer)	Pyradiomics	first-order, shape, GLCM, GLDM GLRLM, GLSZM, NGTDM	RF, SVM, logistic regression	Split sample

NR, not report; RF, random forest; SVM, support vector machine; LLR, LASSO logistics regression model; SE-ResNet, squeeze incentive residual network; GBDT, gradient lifting decision tree; ML, machine learning; CNN, convolution neural network.

**Table 2:** Results of the included studies.

Study ID	TP(T/V)	FP(T/V)	FN(T/V)	TN(T/V)	Sensitivity % (T/V)	Specificity % (T/V)	AUC(Train)	AUC(Validation)
Chang et al. (2021) (15)	145/47	62/12	62/41	140/74	70/53	69/86	0.76 (0.72~0.81)	0.75 (0.68~0.82)
Ruan et al. (2022) (16)	20/10	7/2	10/6	33/12	66.7/62.5	82.5/85.7	0.75	0.62
Wang et al. (2022) (17)	54/17	11/13	6/2	32/17	90.7/89.5	73.9/56.7	0.874(0.81,0.93)	0.786(0.67,0.89)
Mu et al. (2020) (18)	170/68	50/27	31/7	178/85	84.58/90.67	78.07/75.89	0.86 (0.83, 0.90)	0.83 (0.78, 0.89)
Zhang et al. (2020) (25)	66/48	34/34	5/23	68/68	92.8/67.11	66.3/67.04	0.87	0.77
Liu et al. (2020) (20)	16	4	9	5	59.3	83.3	0.73(0.59, 0.87)	—
Huang et al. (2022) (21)	54/28	8/6	10/4	66/19	77/88	91/78	0.90 (0.85–0.95)	0.85 (0.77,0.93)
Zhao et al. (2022) (22)	15/5	9/2	13/4	28/12	53.6/55.6	75.7/85.7	0.727(0.603,0.851)	0.778(0.585,0.970)
Yang et al. (2021) (23)	49	18	4	43	92.5	70.5	0.866(0.799,0.933)	—
Li et al. (2019) (24)	53	11	11	40	82.6	78.3	0.81	—
Zhang et al. (2020) (25)	63/31	14/8	34/5	64/29	64.95/86.11	82.05/78.38	0.79 (0.73,0.86)	0.85 (0.76–0.94)
Wang et al. (2019) (26)	47	12	21	47	69.7	79.1	0.819(0.716, 0.921)	—
Yin et al. (2021) (11)	73/41	15/10	29/10	81/42	71.75/80.39	84.38/80.77	0.86 (0.80-0.91)	0.84 (0.75-0.90)
Nair et al. (2021) (27)	16	4	11	20	78.7	68	0.87	—
Li et al. (2022) (28)	56/23	10/5	17/9	42/17	76.7/71.9	80.8/77.3	0.853 (0.794, 0.905)	0.804 (0.699, 0.898)
Chen et al. (2022) (29)	22	11	15	99	60	90	0.81 ± 0.07	—
Gao et al. (2023) (30)	118/55	75/17	43/15	168/24	73.3/78.6	68.3/58.5	0.76(0.713,0.807)	0.73(0.633,0.828)

T, Training cohort; V, Validation cohort.

### 3.2 Data quality assessment

**Figure 2:**
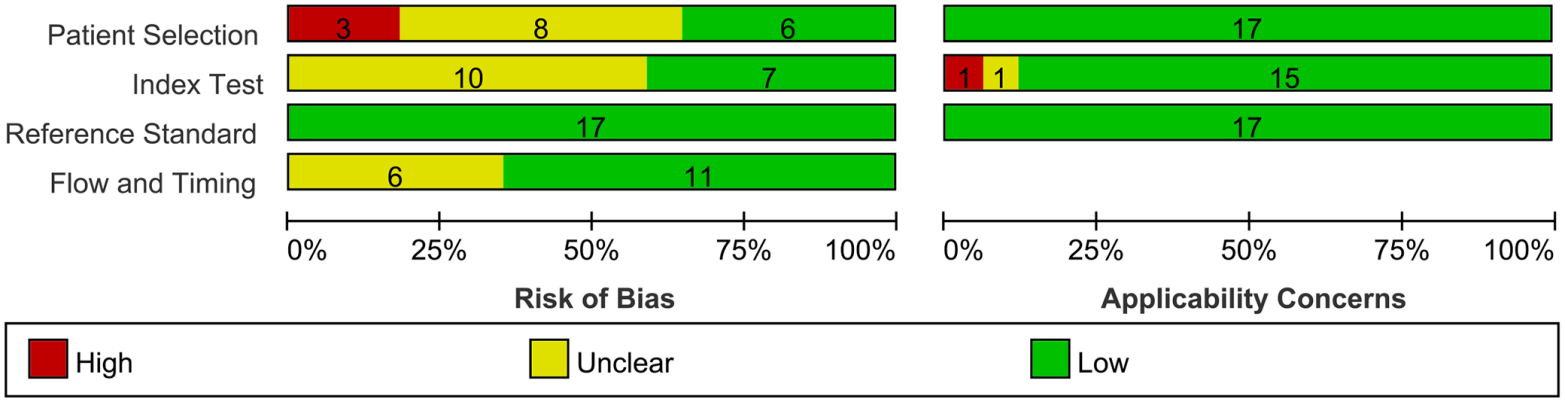
The risk of bias and concerns regarding applicability of included studies.

The results of the QUADAS-2 quality assessment for the literature are shown in **Figure 2**. Due to inappropriate or incomplete exclusion criteria, eleven studies exhibited high or unclear risk of bias in terms of patient selection. Concerning the reference standard, ten studies showed an unclear risk of bias due to missing information on blindness compared to the reference test. Flow and timing introduced uncertainty regarding the risk of bias in six studies, as they exhibited an unclear risk of bias owing to ambiguity in the time interval between the index test and the reference standard. The patient selection of the included studies was of low applicability concern. One study had unclear applicability concerns because the index test was performed with different PET/CT scanners. Another study had high applicability concerns because no information on PET/CT acquisition was provided. Regarding the reference standard, none of the included studies showed an unclear or high risk of bias. Overall, most studies have low or unclear bias risks and moderate clinical applicability problems. The details of the QUADAS-2 assessment are presented in **Figure 2** and **Supplementary Figure S2**.

The included studies achieved a mean ± standard deviation RQS of 10.47 ± 4.72, a median of 12, and a range of 3 to 19. The highest RQS score was 19 (52.8%). RQS scores showed improvement over time (**Supplementary Figure S2**). About half of the studies scored greater than 10. Since no study considered the three items “Phantom study on all scanners”, “Imaging at multiple time points”, and “Prospective study”, these three items received a score of zero. Most studies provided details about the items “Imaging protocol”, “Feature reduction”, “Non Radiomics”, “Biological correlates”, “Discrimination and resampling” and “Gold standard”. The other items that underperformed included “Multiple segmentation,” “Cutoff analysis,” “Calibration statistics,” “Validation,” “Clinical utility,” “Cost-effectiveness analysis,” and “Open science and data,” each with an average score of less than 15%. A detailed description of the RQS scores is provided in **Supplementary Table S6**, **Supplementary Figure S1**. Inter-reviewer agreement for the Radiomics Quality Score (RQS) was quantified using the ICC, which stood at 0.97 (95% CI 0.82-0.99). Across the seven RQS criteria, moderate agreement was observed, while nine items reached substantial or near-perfect concordance, as detailed in **Table 3**.

**Table 3:** Inter-reviewer agreement in RQS assessment.

RQS scoring item	Kappa(95%CI)
Image Protocol	0.46(0.45-0.47)
Multiple Segmentations	1.00(1.00-1.00)
Phantom Study	1.00(1.00-1.00)
Multiple Time Points	1.00(1.00-1.00)
Feature Reduction	0.48(0.47-0.49)
Non Radiomics	0.87(0.50-0.88)
Biological Correlates	0.74(0.73-0.76)
Cut-off	0.67(0.66-0.69)
Discrimination and Resampling	0.77(0.75-0.78)
Calibration	0.76(0.75-0.77)
Prospective	1.00(1.00-1.00)
Validation	0.90(0.88-0.91)
Gold Standard	1.00(1.00-1.00)
Clinical Utility	1.00(1.00-1.00)
Cost-effectiveness	1.00(1.00-1.00)
Open Science	0.57(0.57-0.58)

### 3.3 Meta-analysis combined results

**Figure 3:**
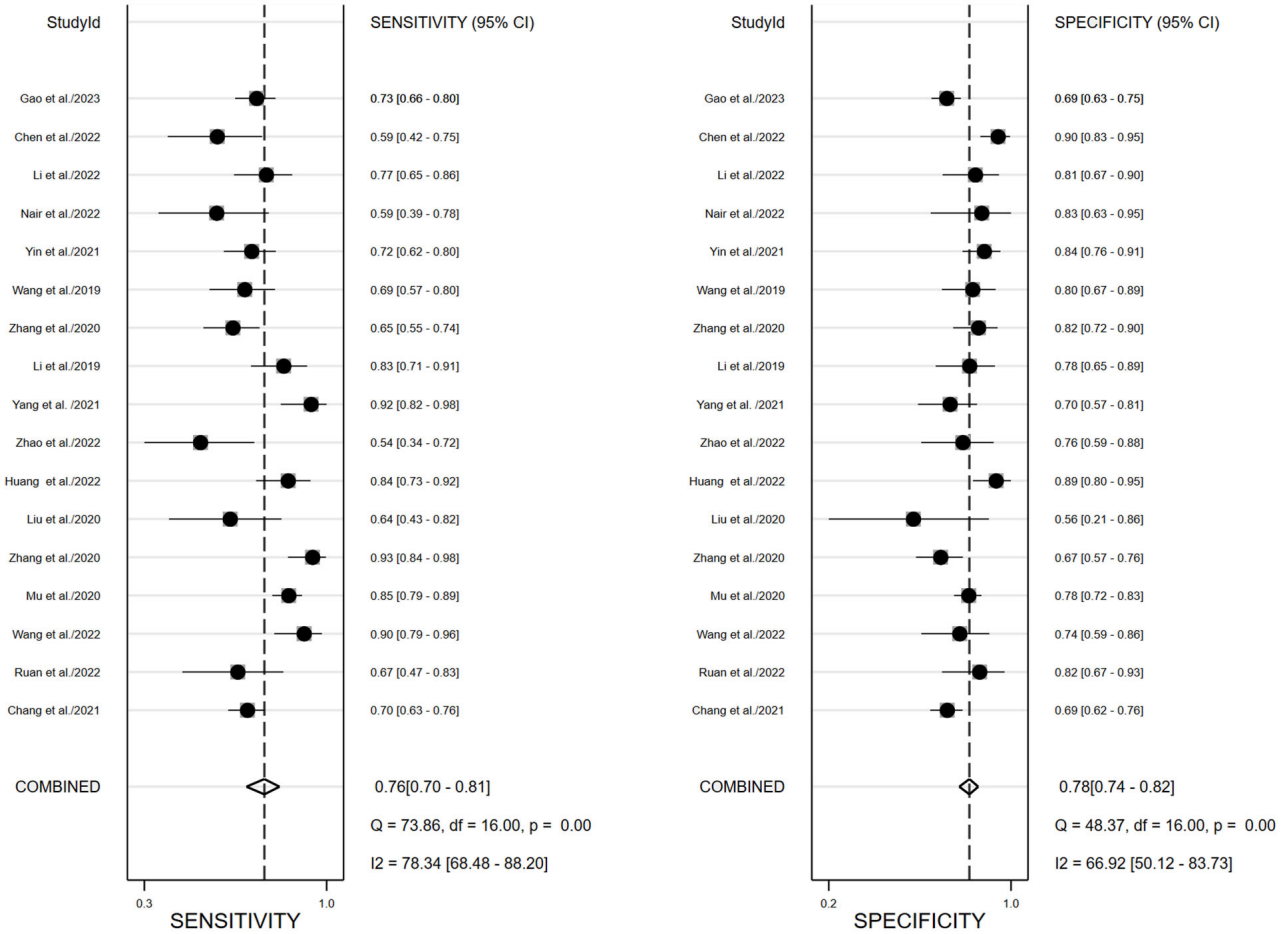
Forest plots of pooled sensitivity and specificity of ^18^F-FDG PET/CT radiomics diagnostic performance of predicting EGFR mutations in NSCLC patients for training cohort.

**Figure 4:**
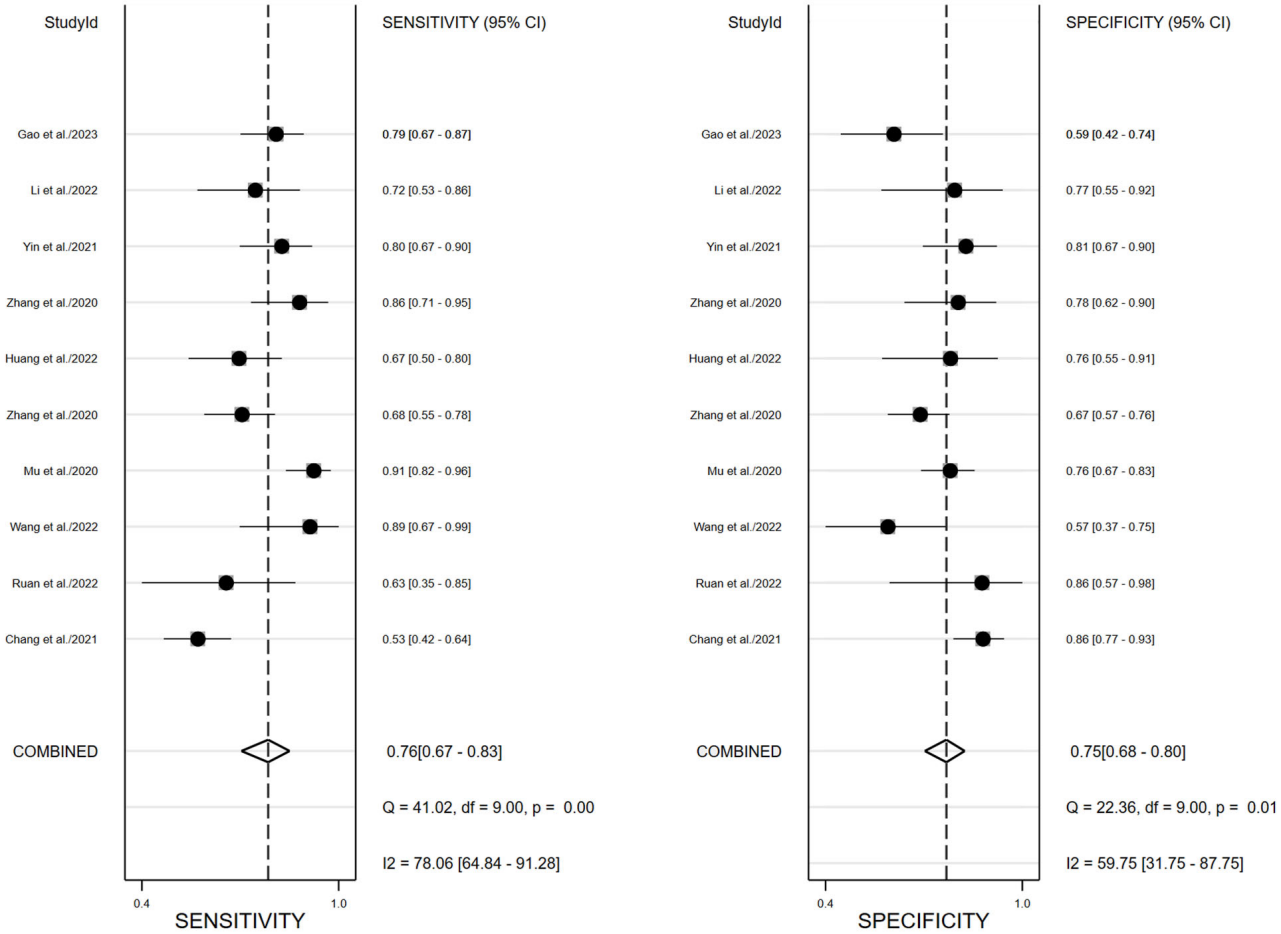
Forest plots of pooled sensitivity and specificity of ^18^F-FDG PET/CT radiomics diagnostic performance of predicting EGFR mutations in NSCLC patients for validation cohort.

**Figure 5:**
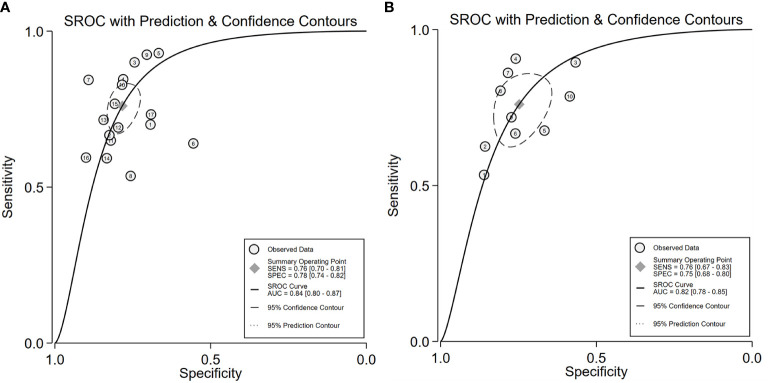
SROC of for the ^18^F-FDG PET/CT radiomics for the prediction of EGFR mutation status in NSCLC, In both training **(A)** and validation cohorts **(B)**.

We performed a meta-analysis to combine the results of the 17 included studies. For train cohort, the pooled SEN, SPE, PLR, NLR, and DOR for the radiomics based on ^18^F-FDG PET/CT in diagnose EGFR mutation status of NSCLC patients were 0.76(0.70,0.81), 0.78(0.74,0.82), 3.5(3.0,4.2), 0.31(0.24,0.39), and 11.0(8.0,16.0) respectively. The forest plot showed significant heterogeneity in sensitivity (*I* ^2 ^= 78.34, *P*<0.01) and specificity (*I^2 ^= *69.92, *P*=0.01). For the validation cohort of 10 studies, the pooled SEN, SPE, PLR, NLR and DOR were 0.76 (0.67,0.83), 0.75 (0.68–0.80), 3.0 (2.4,3.8), 0.32 (0.24,0.44) and 9 (6, 15). **Figures 3**, **4** show the forest plots for the training cohort and validation cohort, respectively. For the training and validation cohorts, the area under the curve (AUC) was 0.84 (95% CI: 0.80-0.87) and 0.82 (95% CI: 0.78-0.85), respectively (**Figures 5A, B**).

### 3.4 Sensitivity analysis and publication bias

**Figure 6:**
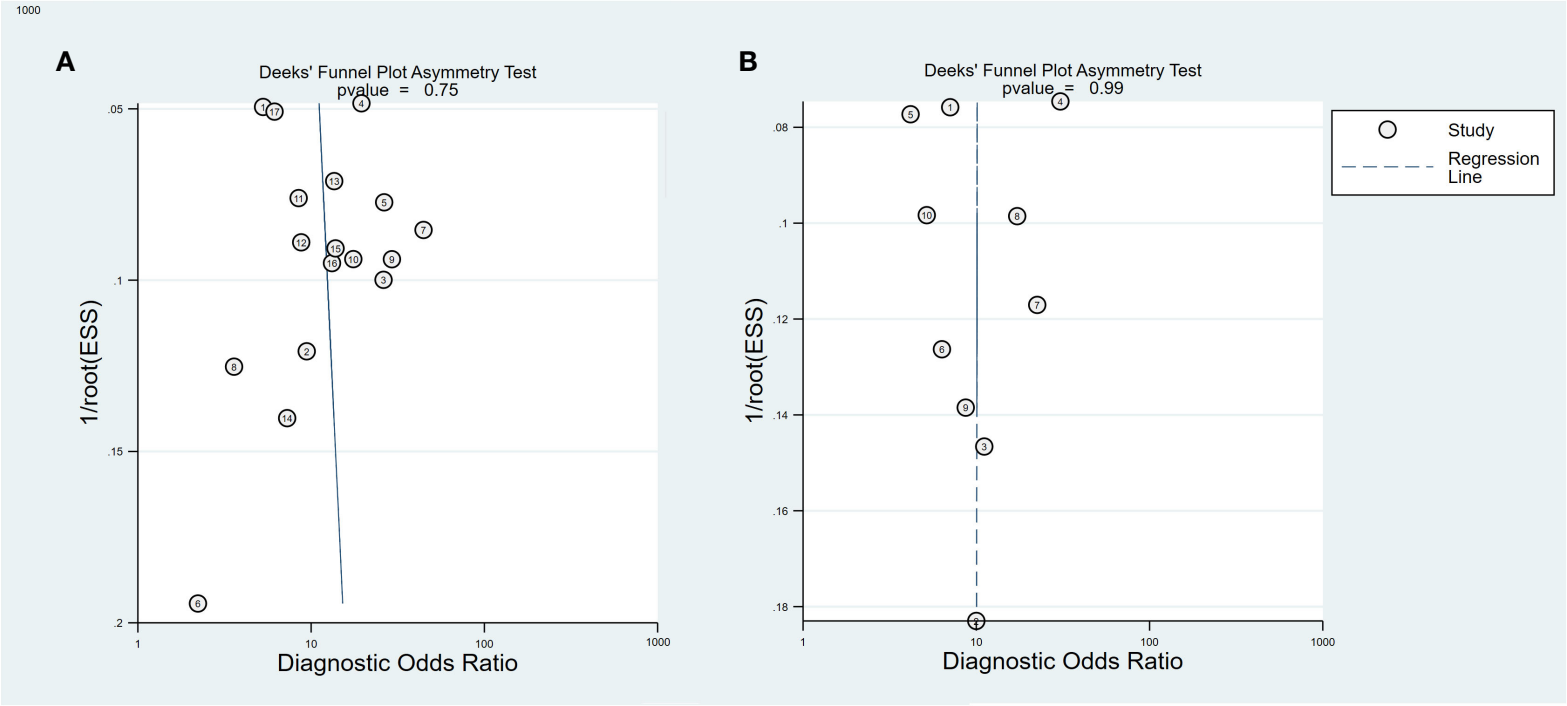
Deeks funnel plot for the publication bias test of ^18^F-FDG PET/CT radiomics for the prediction of EGFR mutation status in NSCLC, In both training **(A)** and validation cohorts **(B)**.

In the included study, the Deek’s test was used to investigate potential publication bias; however, the funnel chart asymmetry test did not show significant publication bias in both training cohorts (t=0.33, p=0.75, **Figure 6A**) or validation t=0.01, p=0.99, **Figure 6B**). We deleted each study individually and combined the rest of the studies to summarize the effect again. The results show that the comprehensive effect of each index changes little, indicating that the stability of the literature is good and the reliability of the results is high (**Supplementary Figure S4**).

### 3.5 Meta-regression subgroup analysis

**Figure 7:**
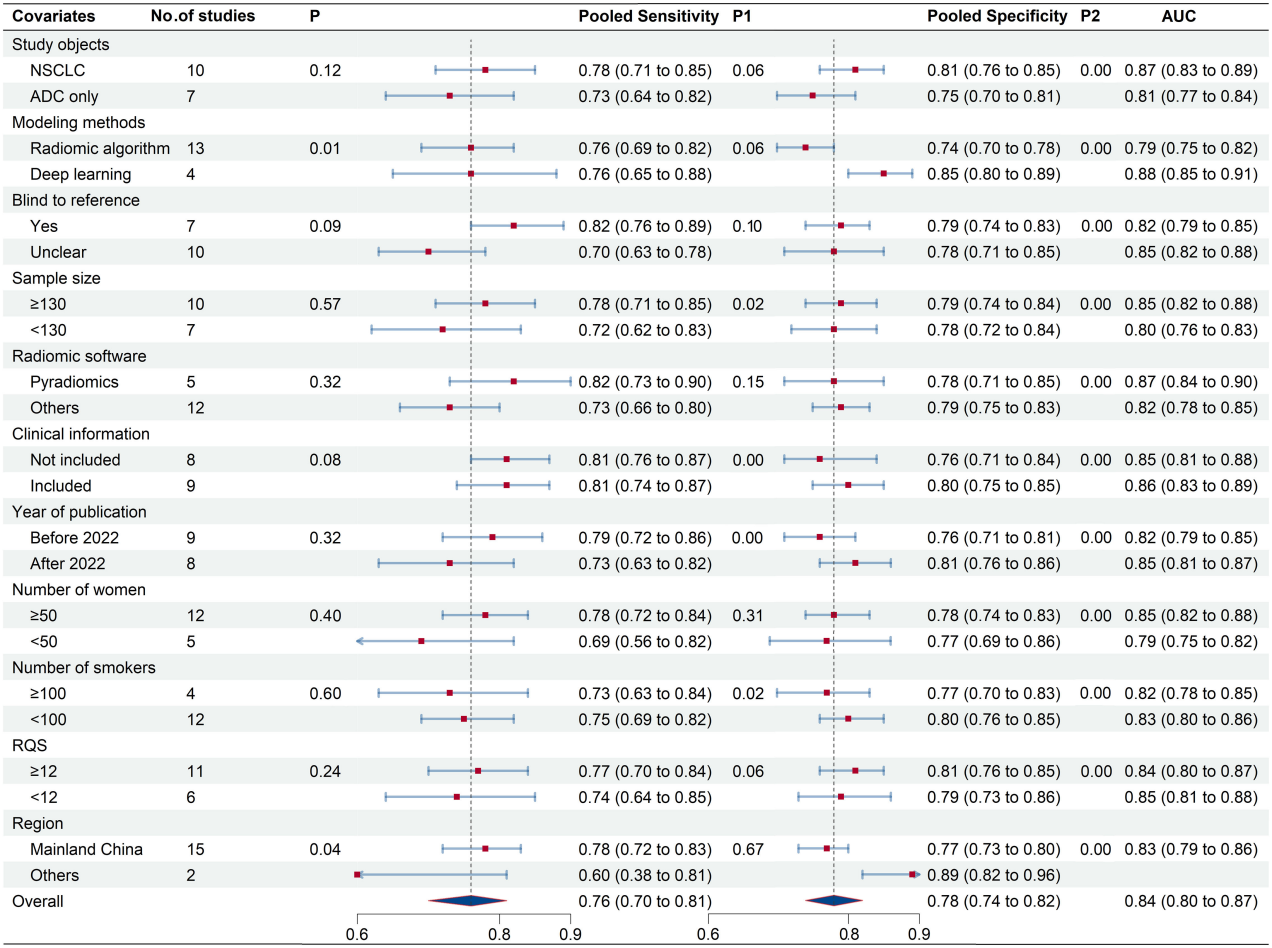
Meta-regression results of ^18^F-FDG PET/CT radiomics for prediction of EGFR mutation in NSCLC.

To elucidate heterogeneity causes, we conducted a meta-regression analysis, detailed in **Figure 7**. Modeling method and region emerged as significant heterogeneity factors, with respective p-values of 0.01 and 0.04. Subgroup analysis indicated that studies (n=10) covering both ADC and other NSCLC subtypes showed enhanced sensitivity (78% vs. 73%, p=0.06) and specificity (81% vs. 75%, p<0.01) compared to ADC-only studies (n=7). Deep learning studies (n=4) outperformed radiomics algorithm studies (n=13) in specificity (85% vs. 74%, p<0.01). Blinded studies (n=7) achieved higher specificity (82% vs. 70%, p=0.10) compared to those with unclear blinding (n=10). Intriguingly, studies utilizing Pyradiomics for feature extraction (n=5) demonstrated superior sensitivity (82% vs. 73%, p=0.15) compared to those employing other software (n=6). Higher RQS studies (n=11) demonstrated increased sensitivity (77%) and specificity (81%) over lower RQS studies (n=6; sensitivity: 74%, p=0.06; specificity: 79%, p<0.01). Larger sample sizes (≥130) were associated with higher sensitivity (78% vs.72%,p=0.57) and specificity (79% vs.78%, p<0.01). Studies with fewer smokers (<100, n=12) outperformed those with more smokers (≥100, n=4) in both sensitivity (75% vs. 73%, p=0.02) and specificity (80% vs. 77%, p<0.01). Similarly, studies with a higher proportion of female participants (n=12) reported better sensitivity (78% vs. 69%, p=0.31) and specificity (78% vs. 77%, p<0.01) than those with fewer females (n=5). Notably, studies integrating clinical data in their radiomics models (n=9) achieved higher specificity (80% vs. 76%, p<0.01) in EGFR mutation prediction.

### 3.6 Clinical utility

**Figure 8:**
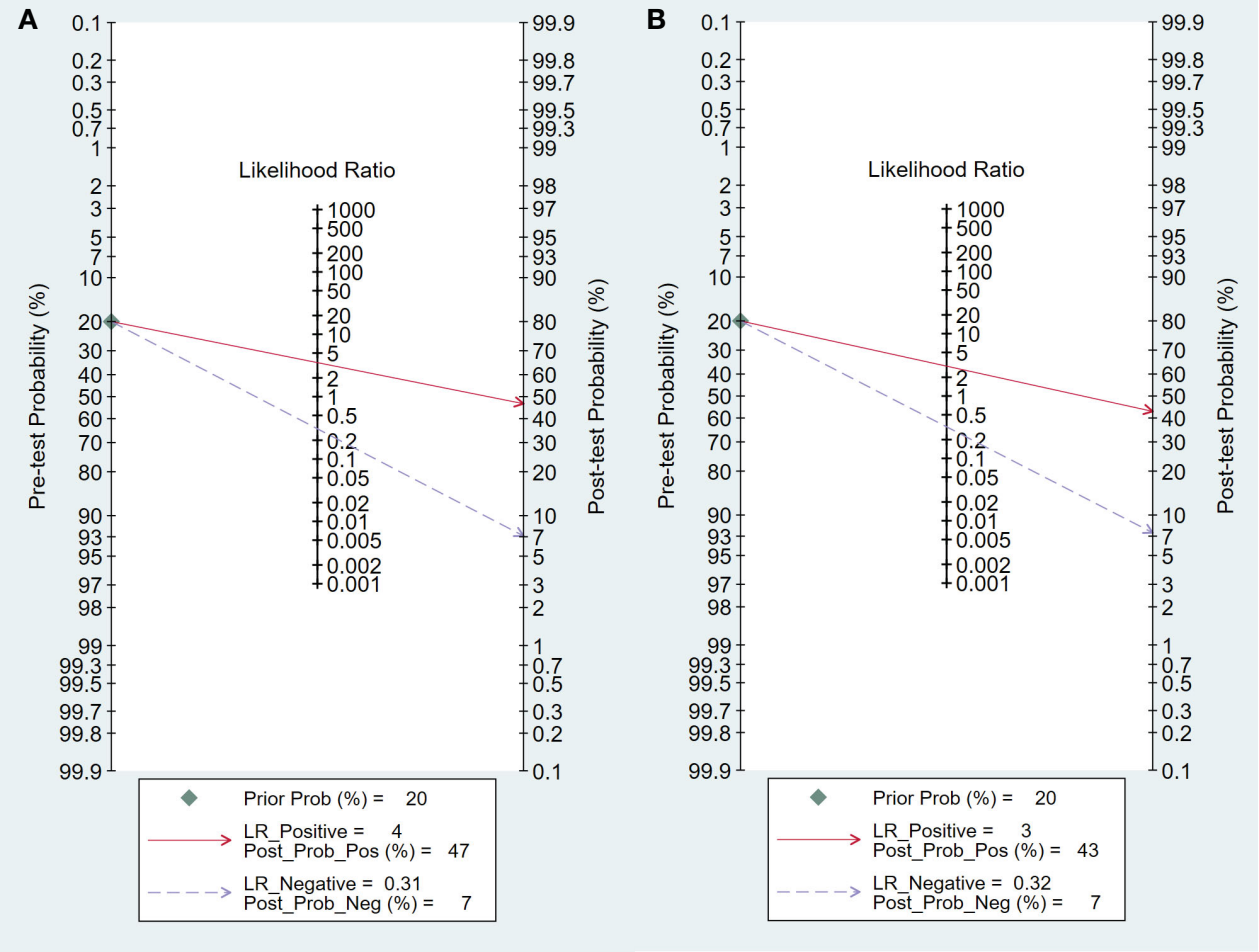
Fagan plots for assessing the clinical utility, In both training **(A)** and validation **(B)** cohorts.

The Fagan plot analysis for the training cohort (**Figure 8A**) demonstrates that ^18^F-FDG PET/CT-based radiomics increased the post-test probability of an EGFR mutation prediction from 20% to 47% with a positive likelihood ratio (PLR) of 5. Conversely, a negative pre-test reduced the post-test probability to 6% with a negative likelihood ratio (NLR) of 0.31. Comparable outcomes were observed in the validation cohort (**Figure 8B**).

## 4 Discussion

EGFR gene is a critical factor in determining the treatment and prognosis of patients with non-small cell lung cancer (NSCLC). EGFR has received increasing attention in recent years as it is frequently overexpressed and directly associated with prolonged survival. EGFR tyrosine kinase inhibitors (TKIs) are more effective in patients with EGFR-mutated NSCLC. Medical imaging techniques such as CT and PET scans are more cost-effective and convenient than biopsies for predicting mutation status. Nguyen et al. first systematic review to the diagnostic accuracy of AI-based radiomics algorithms in predicting EGFR mutation status in lung cancer. The results showed satisfactory diagnostic accuracy with an overall AUC value of 0.789 (31). The combined sensitivity and specificity were 72.2% and 73.3%, respectively. Subgroup analysis revealed that diagnostic performance can be improved by using the radiomics model of PET/CT.

In this review, we detailed synthesize findings from ^18^F-FDG PET/CT radiomics studies targeting EGFR mutation prediction in non-small cell lung cancer. The results of the 17 training cohorts showed that the ^18^F-FDG PET/CT radiomics method was promising for EGFR mutation prediction with a combined sensitivity, specificity, and AUC of 0.74, 0.78, and 0.84, respectively. The corresponding values for the ten independent validation cohorts were 0.76, 0.75, and 0.82, respectively. The results of our study indicated that ^18^F-FDG PET/CT radiomics model can enhance the precision in identifying EGFR mutations in NSCLC patients, aiding clinicians in tailoring treatment regimens, and potentially improving patient outcomes.

The training cohort exhibited significant heterogeneity, necessitating an exploration of its sources. To begin with, threshold effects were considered as they can lead to an overestimation of diagnostic performance. Spearman’s correlation coefficient was evaluated to eliminate the possibility of threshold effects. The results indicated that threshold effects were unlikely to be a source of heterogeneity (*r=0.316, p=0.216*). Besides, in evidence-based medicine, publication bias can significantly influence the results of meta-analysis, potentially leading to distorted or misleading conclusions. We used Deek’s funnel plot to assess publication bias in the included literature. Despite the subjective limitations of Deek’s funnel plot, our observation of a statistically insignificant slope coefficient suggests a low probability of publication bias. We conducted the sensitivity analysis that confirmed the stability of the included literature and the reliability of our findings. Lastly, we analyzed other potential sources of heterogeneity through univariate meta-regression and identified several relevant variables.

Recent systematic evaluations have revealed that the predictive performance of radiomics models in lung cancer is influenced by the inclusion of varying radiomics algorithms and clinical features (31, 32), which explains the heterogeneity of the results based on meta-regression. This variation underpins the heterogeneity observed in our meta-analysis. Notably, in predicting EGFR mutations in lung cancer, deep learning methodologies demonstrate superior performance over conventional machine learning techniques. This enhanced efficacy can be attributed to the advanced capabilities of deep learning models. Unlike traditional machine learning, deep learning can process original imaging data through intricate linear and non-linear transformations utilizing a complex, multi-layer neural network. This approach allows for the extraction of more sophisticated and potentially revealing features from the images. Additionally, deep learning algorithms bypass the need for labor-intensive tumor edge annotation. It can directly learn from the original images, effectively eliminating the requirement for complex preliminary steps such as detailed tumor boundary segmentation, feature extraction, and selection, simplifying the overall process of model development (21). EGFR mutations exhibit a robust association with clinicopathological features, including gender, smoking status, and pathological type. Our subgroup analysis aligns with prior research (33, 34). These results underscore the efficacy of integrating radiomics with clinical data in improving the precision of EGFR mutation predictions. Enriching the model with additional clinical parameters can further elevate diagnostic accuracy of ^18^F-FDG PET/CT radiomics.

Divergences in radiomics feature extraction software can introduce biases in research outcomes. Our subgroup analysis reveals that studies utilizing Pyradiomics for feature extraction demonstrated superior diagnostic efficacy in identifying EGFR mutations, compared to those using alternative software. This variability stems from the distinct algorithmic methodologies and parameter configurations inherent to each software. These findings emphasize the critical influence of feature extraction software selection on study results, underscoring the necessity for clear recognition, comprehension, and transparent reporting of the software’s impact in radiomics research.

Sample size and blinding to reference standards are pivotal in meta-analyses, significantly influencing study quality and contributing to heterogeneity. Adequate sample sizes and reference blinding enhance study reliability and interpretability, while effectively mitigating selection bias. Our analysis indicates that studies blinded to reference standards exhibited notably lower specificity compared to those where blinding status was ambiguous, potentially due to measurement bias in readers aware of the reference standard (8). Furthermore, studies with large sample sizes demonstrated superior diagnostic performance than those with smaller cohorts. Consequently, sample sizes and ensuring blinding to reference standards are critical steps for enhancing the diagnostic accuracy of ^18^F-FDG PET/CT radiomics in identifying EGFR mutations.

The dynamic progression of study typically places greater emphasis on recent studies for their applicability to contemporary clinical practice. In our subgroup analysis, studies published after 2022 show improved predictive accuracy for EGFR mutations, possibly due to advancements in imaging equipment, radiomics algorithms, and enhanced study quality. (35). Recognizing and addressing these temporal variations is crucial for deriving precise and relevant meta-analysis conclusions.

The quality of the studies included in this review was assessed using QUADAS-2 and RQS. QUADAS-2 was used for diagnostic accuracy studies, while RQS was used for quality assessment of radiomics studies. QUADAS-2 revealed high or unclear risks in reference standards, flow, and timing in many studies. Over two-thirds had unclear risks due to non-specifics about participant sampling methods, and three studies showed high selection bias risk from inappropriate exclusions. The timing of ^18^F-FDG PET/CT relative to biopsy, a crucial factor in flow and timing domains, was often ambiguously reported, leading to unclear temporal domain risks. Nevertheless, patient selection, index testing, and reference standards generally had low applicability concerns, indicating a broadly acceptable quality of the included studies.

It is similar to recent systematic evaluations of the quality of radiomics studies in other fields, the median RQS score of included studies was 12 (33.3% of the total score), which is overall relatively low (31, 36). In radiomics research, the RQS critically influences study results. Studies that garner low RQS ratings are often plagued by methodological defects. These deficiencies can introduce significant biases into the meta-analysis, consequently diminishing the reliability and validity of the research findings. The methodological rigor of the included studies was inconsistent. A notable absence of prospective designs and, often, multicenter independent validation cohorts hindered the assessment of model reproducibility. Furthermore, limited biological relevance in investigations and a scarcity of publicly open data could impede a thorough understanding of how ^18^F-FDG PET/CT radiomic features influence EGFR prediction. Our meta-regression found no significant link between the RQS and result heterogeneity. While study quality metrics are important, low RQS scores should be interpreted as indicators of areas for improvement rather than outright poor quality. Notably, deep learning studies may be disadvantaged by RQS, which is more suited to hand-crafted radiomics (21), indicating RQS might not be the optimal tool for radiomics quality assessment. In contrast, new checklists like CLEAR have shown efficacy in reporting radiomics modeling components, which provides a more thorough coverage of study aspects, enhancing the comprehensive evaluation of study quality and reliability in radiomics research (37).

This study has several limitations. Predominantly, the population analyzed was Chinese, with only two of the 17 studies based in the USA and Canada, while the rest were conducted in China. This led to significant geographical variations contributing to the heterogeneity of our results. EGFR mutations were found in 15.4% of North American NSCLC patients, in contrast to 49.1% in Asian patients, highlighting substantial regional differences (38). Moreover, the retrospective nature of all included studies underscores the need for prospective research to enhance findings’ quality and relevance. Additionally, the exclusion of gray literature and studies with inadequate data may result in biased meta-analysis, necessitating careful consideration in study selection and analysis to ensure the validity and accuracy of the results.

In conclusion, our meta-analysis demonstrates that ^18^F-FDG PET/CT radiomics is a promising tool for predicting EGFR mutations in NSCLC. Deep learning algorithms particularly stand out, offering enhanced predictive accuracy. However, the pooled AUCs for both validation and training cohorts in our study fall below 0.90, suggesting that this field is still in its developmental phase. This early stage of research limits the wider clinical application of these noninvasive methods for EGFR mutation assessment. Therefore, the development of more advanced deep learning features based on ^18^F-FDG PET/CT is essential for improving the predictive accuracy for EGFR mutations in NSCLC.

## Data availability statement

The original contributions presented in the study are included in the article/**Supplementary Material**, further inquiries can be directed to the corresponding author.

## Author contributions

NM: Conceptualization, Methodology, Writing – original draft, Writing – review & editing. WY: Writing – original draft, Data curation. QW: Data curation, Writing – review & editing. CC: Writing – review & editing, Methodology. YH: Writing – review & editing. ZW: Funding acquisition, Supervision, Writing – review & editing.
