# Supplementary material for: Predictive value of 18F-FDG PET/CT radiomics for EGFR mutation status in non-small cell lung cancer: a systematic review and meta-analysis
Source: Front Oncol. 2024 Feb 1;14:1281572. doi: 10.3389/fonc.2024.1281572 (PMC10867100; doi:10.3389/fonc.2024.1281572)
Supplement: Supplementary file 1 [file DataSheet_1.pdf]

Supplementary Material

Predictive Value of ^{18}F-FDG PET/CT Radiomics for EGFR Mutation Status in Non-Small Cell Lung Cancer: A systematic review and meta-analysis

Ning Ma[1], Weihua Yang[1], Qiannan Wang[1], Caozhe CUI[1], Yiyi HU[1], Zhifang Wu[1,2*]

*** Correspondence:** Zhifang Wu
wuzhifang01@163.com

Supplementary Figures and Tables

Supplementary Tables

Table S1. PubMed searching algorithm

#1	Lung Neoplasms[MeSH Terms]
#2	Search (((((((((((((((((((((Pulmonary Neoplasms) OR (Neoplasms, Lung)) OR (Lung Neoplasm)) OR (Neoplasm, Lung)) OR (Neoplasms, Pulmonary)) OR (Neoplasm, Pulmonary)) OR (Pulmonary Neoplasm)) OR (Lung Cancer)) OR (Cancer, Lung)) OR (Cancers, Lung)) OR (Lung Cancers)) OR (Pulmonary Cancer)) OR (Cancer, Pulmonary)) OR (Cancers, Pulmonary)) OR (Pulmonary Cancers)) OR (Cancer of the Lung)) OR (Cancer of Lung)) OR (Lung Adenocarcinomas)) OR (Lung Adenocarcinoma)) OR (Adenocarcinoma, Lung)) OR (Adenocarcinomas, Lung)) OR (Adenocarcinoma of Lung)
#3	#1 OR #2
#4	ErbB Receptors[MeSH Terms]
#5	(((((((((((((((((((((((((((((((((((((Receptors, ErbB) OR (Transforming Growth Factor alpha Receptor)) OR (Urogastrone Receptor)) OR (Receptor, TGF-alpha)) OR (Receptor, TGF alpha)) OR (Epidermal Growth Factor Receptor Kinase)) OR (Epidermal Growth Factor Receptor Protein-Tyrosine Kinase)) OR (Epidermal Growth Factor Receptor Protein Tyrosine Kinase)) OR (Receptor, Urogastrone)) OR (Receptor, Transforming-Growth Factor alpha)) OR (Receptor, Transforming Growth Factor alpha)) OR (TGF-alpha Receptor)) OR (HER Family Receptors)) OR (Family Receptors, HER)) OR (Receptors, HER Family)) OR (Receptor, ErbB-1)) OR (ErbB-1 Receptor)) OR (Receptor, ErbB 1)) OR (Proto-oncogene c-ErbB-1 Protein)) OR (Proto oncogene c ErbB 1 Protein)) OR (c-ErbB-1 Protein, Proto-oncogene)) OR (c-erbB-1 Protein)) OR (c erbB 1 Protein)) OR (Receptor Tyrosine-protein Kinase erbB-1)) OR (Receptor Tyrosine protein Kinase erbB 1)) OR (erbB-1 Proto-Oncogene Protein)) OR (Proto-Oncogene Protein, erbB-1)) OR (erbB 1 Proto Oncogene Protein)) OR (EGF Receptors)) OR (Receptors, EGF)) OR (Epidermal Growth Factor Receptor)) OR (Receptors, Epidermal Growth Factor-Urogastrone)) OR (Receptors, Epidermal Growth Factor Urogastrone)) OR (Receptor, EGF)) OR (Receptors, Epidermal Growth Factor)) OR (Epidermal Growth Factor Receptor Family Proteins)) OR (EGF Receptor)) OR (Receptor, Epidermal Growth Factor)
#6	#4 OR #5
#7	Positron-Emission Tomography[MeSH Terms]
#8	((((((Positron Emission Tomography) OR (PET Scan)) OR (PET Scans)) OR (Scan, PET)) OR (Scans, PET)) OR (Tomography, Positron-Emission)) OR (Tomography, Positron Emission)
#9	#7 OR #8
#10	Fluorodeoxyglucose F18[MeSH Terms]
#11	((((((((((((((F18, Fluorodeoxyglucose) OR (18F-FDG)) OR (Fluorodeoxyglucose F 18)) OR (F 18, Fluorodeoxyglucose)) OR (Fludeoxyglucose F 18)) OR (F 18, Fludeoxyglucose)) OR (Fluorine-18-fluorodeoxyglucose)) OR (Fluorine 18 fluorodeoxyglucose)) OR (18F Fluorodeoxyglucose)) OR (Fluorodeoxyglucose, 18F)) OR (18FDG)) OR (2-Fluoro-2-deoxy-D-glucose)) OR (2 Fluoro 2 deoxy D glucose)) OR (2-Fluoro-2-deoxyglucose)) OR (2 Fluoro 2 deoxyglucose)
#12	#10 OR #11
#13	((((((textural*) OR (texture*)) OR (radiomics*)) OR (radiomic*)) OR (radiogenomic*)) OR (machine learning)) OR (Artificial Intelligence)
#14	#3 AND #6 AND #9 AND #12 AND #13

Table S2. Embase searching algorithm

#1	'lung cancer'/exp
#2	'pulmonary neoplasms' OR 'neoplasms, lung' OR 'lung neoplasm' OR 'neoplasm, lung' OR 'neoplasms, pulmonary' OR 'neoplasm, pulmonary' OR 'pulmonary neoplasm' OR 'lung cancer' OR 'cancer, lung' OR 'cancers, lung' OR 'lung cancers' OR 'pulmonary cancer' OR 'cancer, pulmonary' OR 'cancers, pulmonary' OR 'pulmonary cancers' OR 'cancer of the lung' OR 'cancer of lung' OR 'lung adenocarcinomas' OR 'lung adenocarcinoma' OR 'adenocarcinoma, lung' OR 'adenocarcinomas, lung'
#3	#1 OR #2
#4	'epidermal growth factor receptor'/exp
#5	'receptors, erbb' OR 'transforming growth factor alpha receptor' OR 'urogastrone receptor' OR 'receptor, tgf-alpha' OR 'receptor, tgf alpha' OR 'epidermal growth factor receptor kinase' OR 'epidermal growth factor receptor protein-tyrosine kinase' OR 'epidermal growth factor receptor protein tyrosine kinase' OR 'receptor, urogastrone' OR 'receptor, transforming-growth factor alpha' OR 'receptor, transforming growth factor alpha' OR 'tgf-alpha receptor' OR 'her family receptors' OR 'family receptors, her' OR 'receptors, her family' OR 'receptor, erbb-1' OR 'erbb-1 receptor' OR 'receptor, erbb 1' OR 'proto-oncogene c-erbb-1 protein' OR 'proto oncogene c erbb 1 protein' OR 'c-erbb-1 protein, proto-oncogene' OR 'c-erbb-1 protein' OR 'c erbb 1 protein' OR 'receptor tyrosine-protein kinase erbb-1' OR 'receptor tyrosine protein kinase erbb 1' OR 'erbb-1 proto-oncogene protein' OR 'proto-oncogene protein, erbb-1' OR 'erbb 1 proto oncogene protein' OR 'egf receptors' OR 'receptors, egf' OR 'epidermal growth factor receptor' OR 'receptors, epidermal growth factor-urogastrone' OR 'receptors, epidermal growth factor urogastrone' OR 'receptor, egf' OR 'receptors, epidermal growth factor' OR 'epidermal growth factor receptor family proteins' OR 'egf receptor' OR 'receptor, epidermal growth factor'
#6	#4 OR #5
#7	'positron emission tomography'/exp
#8	'positron emission tomography' OR 'pet scan' OR 'pet scans' OR 'scan, pet' OR 'scans, pet' OR 'tomography, positron-emission' OR 'tomography, positron emission' OR 'positron-emission tomography'
#9	#7 OR #8
#10	'fluorodeoxyglucose f 18'/exp
#11	'f18, fluorodeoxyglucose' OR '18f-fdg' OR 'fluorodeoxyglucose f 18' OR 'f 18, fluorodeoxyglucose' OR 'fludeoxyglucose f 18' OR 'f 18, fludeoxyglucose' OR 'fluorine-18-fluorodeoxyglucose' OR 'fluorine 18 fluorodeoxyglucose' OR '18f fluorodeoxyglucose' OR 'fluorodeoxyglucose, 18f' OR '18fdg' OR '2-fluoro-2-deoxy-d-glucose 2 fluoro 2 deoxy d glucose' OR '2-fluoro-2-deoxyglucose' OR '2 fluoro 2 deoxyglucose'
#12	#10 OR #11
#13	'textural'* OR 'texture*' OR 'radiomics*' OR 'radiomic*' OR 'radiogenomic*' OR 'machine learning' OR 'Artificial Intelligence'
#13	#3 AND #6 AND #9 AND #12 AND #13

Table S3. Web of Science searching algorithm

#1	TS=(Lung Neoplasm* OR Neoplasm*, Lung OR Neoplasm*, Pulmonary OR Pulmonary Neoplasm* OR Lung Cancer* OR Cancer, Lung* OR Pulmonary Cancer* OR Cancer*, Pulmonary OR Cancer* of the Lung OR Cancer* of Lung OR Lung Adenocarcinoma* OR Adenocarcinoma*, Lung OR Adenocarcinoma of Lung)
#2	TS=(ErbB Receptors OR Receptors, ErbB OR Transforming Growth Factor alpha Receptor OR Urogastrone Receptor OR Receptor, TGF-alpha OR Receptor, TGF alpha OR Epidermal Growth Factor Receptor Kinase OR Epidermal Growth Factor Receptor Protein-Tyrosine Kinase OR Epidermal Growth Factor Receptor Protein Tyrosine Kinase OR Receptor, Urogastrone OR Receptor, Transforming-Growth Factor alpha OR Receptor, Transforming Growth Factor alpha OR TGF-alpha Receptor OR HER Family Receptors OR Family Receptors, HER OR Receptors, HER Family OR Receptor, ErbB-1 OR ErbB-1 Receptor OR Receptor, ErbB 1 OR Proto-oncogene c-ErbB-1 Protein OR Proto oncogene c ErbB 1 Protein OR c-ErbB-1 Protein, Proto-oncogene OR c-erbB-1 Protein OR c erbB 1 Protein OR Receptor Tyrosine-protein Kinase erbB-1 OR Receptor Tyrosine protein Kinase erbB 1 OR erbB-1 Proto-Oncogene Protein OR Proto-Oncogene Protein, erbB-1 OR erbB 1 Proto Oncogene Protein OR EGF Receptors OR Receptors, EGF OR Epidermal Growth Factor Receptor OR Receptors, Epidermal Growth Factor-Urogastrone OR Receptors, Epidermal Growth Factor Urogastrone OR Receptor, EGF OR Receptors, Epidermal Growth Factor OR Epidermal Growth Factor Receptor Family Proteins OR EGF Receptor OR Receptor, Epidermal Growth Factor)
#3	TS=(Positron-Emission Tomography OR Positron Emission Tomography OR PET Scan* OR Scan*, PET OR Tomography, Positron-Emission OR Tomography, Positron Emission)
#4	TS=(Fluorodeoxyglucose F18 OR F18, Fluorodeoxyglucose OR 18F-FDG OR Fluorodeoxyglucose F 18 OR F 18, Fluorodeoxyglucose OR Fludeoxyglucose F 18 OR F 18, Fludeoxyglucose OR Fluorine-18-fluorodeoxyglucose OR Fluorine 18 fluorodeoxyglucose OR 18F Fluorodeoxyglucose OR Fluorodeoxyglucose, 18F OR 18FDG OR 2-Fluoro-2-deoxy-D-glucose OR 2 Fluoro 2 deoxy D glucose OR 2-Fluoro-2-deoxyglucose OR 2 Fluoro 2 deoxyglucose)
	TS=(textural* OR texture* OR radiomics* OR radiomic* OR radiogenomic* OR machine learning OR Artificial Intelligence)
#5	#4 AND #3 AND #2 AND #1

Table S4. Cochrane searching algorithm

#1	MeSH descriptor: [Lung Neoplasms] explode all trees
#2	MeSH descriptor: [Adenocarcinoma of Lung] explode all trees
#3	(Lung Neoplasm* OR Neoplasm*, Lung OR Neoplasm*, Pulmonary OR Pulmonary Neoplasm* OR Lung Cancer* OR Cancer, Lung* OR Pulmonary Cancer* OR Cancer*, Pulmonary OR Cancer* of the Lung OR Cancer* of Lung OR Lung Adenocarcinoma* OR Adenocarcinoma*, Lung OR Adenocarcinoma of Lung):ti,ab,kw
#4	#1 OR #2 OR #3
#5	MeSH descriptor: [ErbB Receptors] explode all trees
#6	(ErbB Receptors OR Receptors, ErbB OR Transforming Growth Factor alpha Receptor OR Urogastrone Receptor OR Receptor, TGF-alpha OR Receptor, TGF alpha OR Epidermal Growth Factor Receptor Kinase OR Epidermal Growth Factor Receptor Protein-Tyrosine Kinase OR Epidermal Growth Factor Receptor Protein Tyrosine Kinase OR Receptor, Urogastrone OR Receptor, Transforming-Growth Factor alpha OR Receptor, Transforming Growth Factor alpha OR TGF-alpha Receptor OR HER Family Receptors OR Family Receptors, HER OR Receptors, HER Family OR Receptor, ErbB-1 OR ErbB-1 Receptor OR Receptor, ErbB 1 OR Proto-oncogene c-ErbB-1 Protein OR Proto oncogene c ErbB 1 Protein OR c-ErbB-1 Protein, Proto-oncogene OR c-erbB-1

	Protein OR c erbB 1 Protein OR Receptor Tyrosine-protein Kinase erbB-1 OR Receptor Tyrosine protein Kinase erbB 1 OR erbB-1 Proto-Oncogene Protein OR Proto-Oncogene Protein, erbB-1 OR erbB 1 Proto Oncogene Protein OR EGF Receptors OR Receptors, EGF OR Epidermal Growth Factor Receptor OR Receptors, Epidermal Growth Factor-Urogastrone OR Receptors, Epidermal Growth Factor Urogastrone OR Receptor, EGF OR Receptors, Epidermal Growth Factor OR Epidermal Growth Factor Receptor Family Proteins OR EGF Receptor OR Receptor, Epidermal Growth Factor):ti,ab,kw
#7	#5 OR #6
#8	MeSH descriptor: [Positron-Emission Tomography] explode all trees
#9	(Positron Emission Tomography OR PET Scan* OR Scan*, PET OR Tomography, Positron-Emission OR Tomography, Positron Emission):ti,ab,kw
#10	#8 OR #9
#11	(textural* OR texture* OR radiomics* OR radiomic* OR radiogenomic* OR machine learning OR Artificial Intelligence) :ti,ab,kw
#11	#4 AND #7 AND #10

Table S5. China National Knowledge Infrastructure searching algorithm

	Title	Lung cancer + non-small cell lung cancer + bronchogenic carcinoma + primary bronchogenic carcinoma + bronchogenic carcinoma + NSCLC
AND	Title	Epidermal growth factor receptor + human epidermal growth factor receptor + epidermal growth factor receptor + EGFR
AND	Title	PET + "PET/CT" + positron emission computed tomography + FDG + "18F-FDG" +Fluoroglucose
AND	Title	Radiomics + Texture analysis + Machine learning + Deep learning + Radiogenomics

Table S6. RQS Ratings and average rating per item

Study ID	Image Protocol	Multiple Segmentations	Phantom Study	Multiple Time Points	Feature Reduction	Non Radiomics	Biological Correlates	Cut-off	Discrimination and Resampling	Calibration	Prospective	Validation	Gold Standard	Clinical Utility	Cost-effectiveness	Open Science	Total
Chang et al. (2021)	1/1/-	1/1/-	0/0/-	0/0/-	3/3/-	0/0/-	1/1/-	1/1/-	2/2/-	0/0/-	0/0/-	2/2/-	2/2/-	0/0/-	1/1/-	0/0/-	14/14
Ruan et al. (2022)	1/1/-	1/1/-	0/0/-	0/0/-	3/3/-	1/1/-	1/1/-	0/0/-	1/1/1	0/0/-	0/0/-	2/2/-	2/2/-	0/0/-	0/0/-	0/0/-	12/12
Wang et al. (2021)	1/1/-	1/1/-	0/0/-	0/0/-	3/3/-	1/1/-	1/0/0	0/0/-	1/1/1	1/1/-	0/0/-	2/2/-	2/2/-	2/2/-	0/0/-	0/0/-	15/14
Mu et al. (2020)	1/0/0	0/0/-	0/0/-	0/0/-	3/3/-	1/1/-	1/1/-	1/1-	2/1/1	0/0/-	0/0/-	5/5/-	2/2/-	0/0/-	0/0/-	3/2/2	19/17
Zhang et al. (2020)	1/1/-	0/0/-	0/0/-	0/0/-	3/3/-	1/0/0	1/1/-	0/0/-	2/2/-	1/1/-	0/0/-	-5/-5/-	2/2/-	0/0/-	0/0/-	0/0/-	6/5
Liu et al. (2020)	1/1/-	1/1/-	0/0/-	0/0/-	3/3/-	0/0/-	0/0/-	1/1/-	1/1/-	0/0/-	0/0/-	-5/-5/-	2/2/-	0/0/-	0/0/-	0/0/-	4/4

Huang et al. (2022)	1/1/-	0/0/-	0/0/-	0/0/-	3/3/-	1/1/-	1/1/-	0/0/-	1/1/-	1/1/-	0/0/-	2/2/-	2/2/-	0/0/-	0/0/-	0/0/-	12/12
Zhao et al. (2022)	1/1/-	0/0/-	0/0/-	0/0/-	3/3/-	1/1/-	0/0/-	0/0/-	1/1/-	1/1/-	0/0/-	2/2/-	2/2/-	2/2/-	0/0/-	0/0/-	13/13
Yang et al. (2021)	1/1/-	0/0/-	0/0/-	0/0/-	3/3/-	0/0/-	0/0/-	0/0/-	2/2/-	2/1/1	0/0/-	2/2/-	2/2/-	0/0/-	0/0/-	0/0/-	12/11
Li et al. (2019)	1/1/-	1/1/-	0/0/-	0/0/-	3/3/-	0/0/-	1/1/-	0/0/-	1/1/-	0/0/-	0/0/-	-5/-5/-	2/2/-	0/0/-	0/0/-	0/0/-	6/6
Zhang et al. (2020)	1/1/-	1/1/-	0/0/-	0/0/-	3/3/-	1/1/-	1/0/0	0/0/-	1/1/-	0/0/-	0/0/-	2/2/-	2/2/-	0/0/-	0/0/-	0/0/-	12/11
Wang et al. (2019)	1/1/-	1/1/-	0/0/-	0/0/-	3/3/-	1/1/-	0/0/0	0/0/-	1/1/-	0/0/-	0/0/-	-5/-5/-	2/2/-	0/0/-	0/0/-	0/0/-	4/6
Yin et al. (2021)	1/1/-	1/1/-	0/0/-	0/0/-	3/3/-	1/1/-	1/1/-	0/0/-	1/1/-	0/0/-	0/0/-	2/2/-	22/2/-	0/0/-	0/0/-	0/0/-	12/11
Nair et al. (2021)	1/1/-	0/0/-	0/0/-	0/0/-	3/3/-	0/0/-	0/0/-	0/0/-	2/2/-	0/0/-	0/0/-	-5/-5/-	2/2/-	0/0/-	0/0/-	0/0/-	3/4
Li et al. (2022)	1/1/-	1/1/-	0/0/-	0/0/-	3/3/-	1/1/-	1/1/-	0/1/1	1/0/0	0/0/-	0/0/-	2/2/-	2/2/-	0/0/-	0/0/-	0/0/-	12/12

Chen et al. (2022)	1/0/0	0/0/-	0/0/-	0	3/0/0	1/1/-	1/1/-	0/0/-	2/2/0	0/0/-	0/0/-	-5/-5/-	2/2/-	0/0/-	0/0/-	0/1/1	5/3
Gao et al. (2023)	1/1/-	0/0/-	0/0/-	0	3/3/-	1/1/-	1/1/-	1/0/0	2/2/0	2/1/1	0/0/-	2/3/3	2/2/-	2/2/-	0/0/-	0/0/-	17/16
Define d range	0-2	0-1	0-1	0-1	-3-3	0-1	0-1	0-1	0-2	0-1	0-7	-5-5	0-2	0-2	0-1	0-4	-8-36

Supplementary Figures

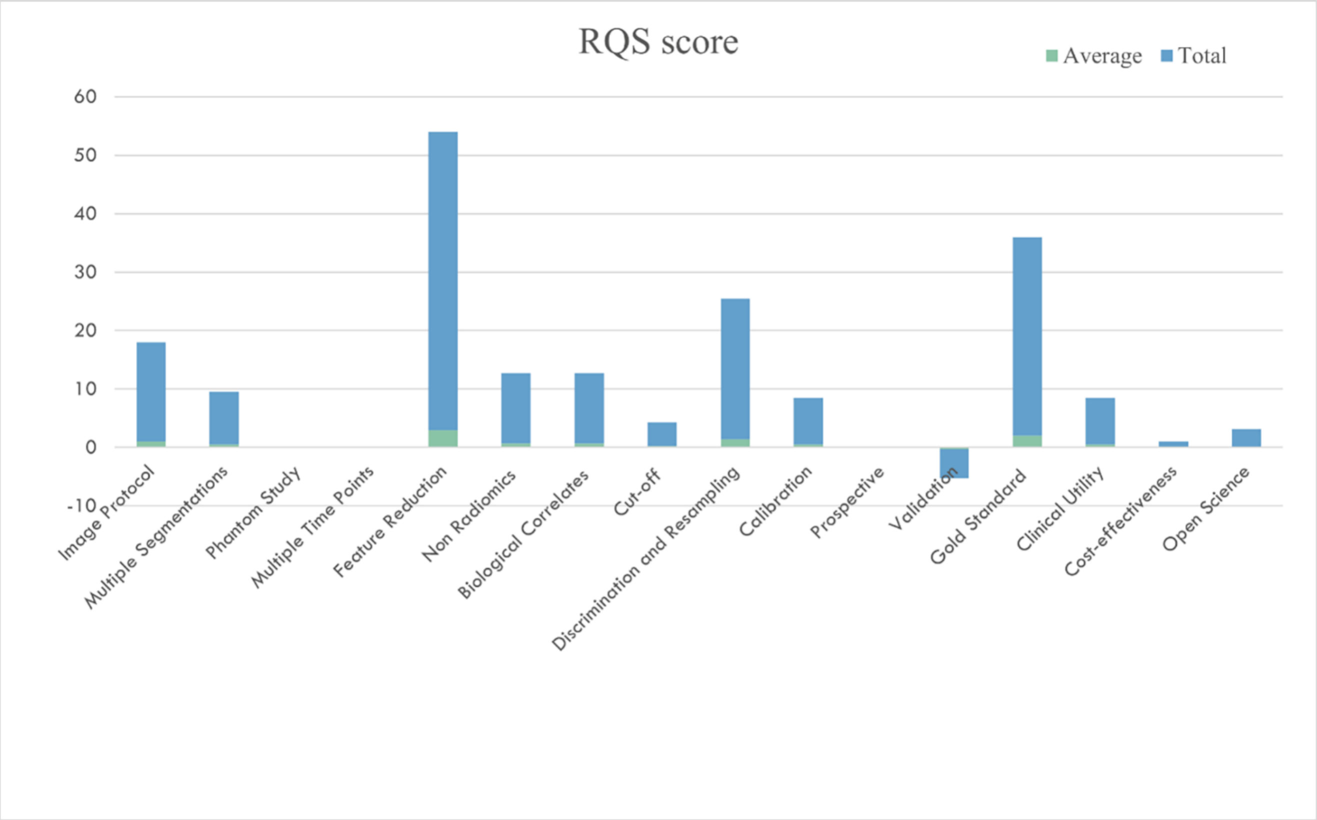

Figure S1 Methodological quality evaluated by using the Radiomics Quality Score (RQS) tool. Average scores of each RQS item

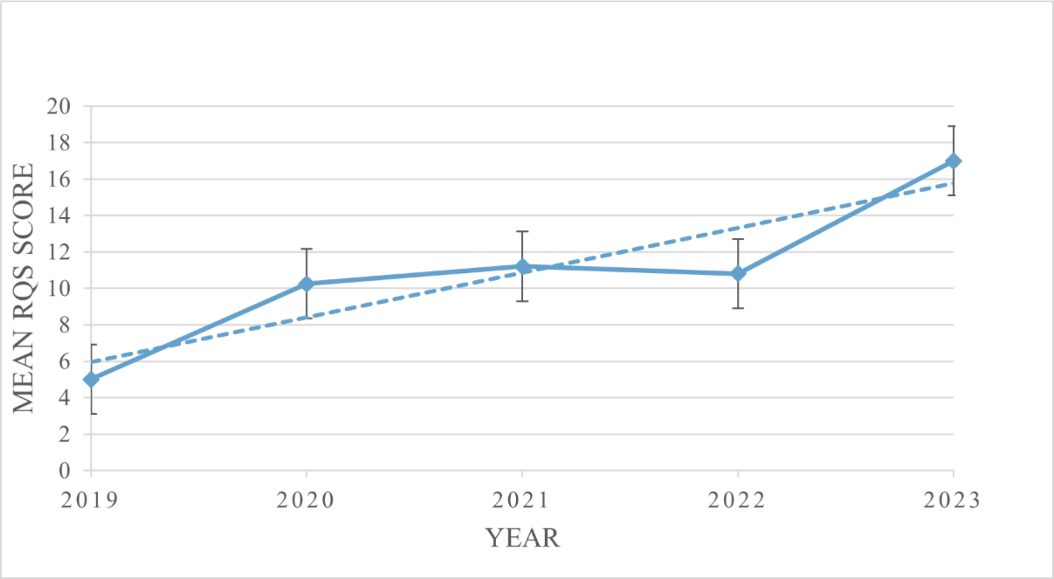

Figure S2 Yearly trends in the average RQS Score of the included studies

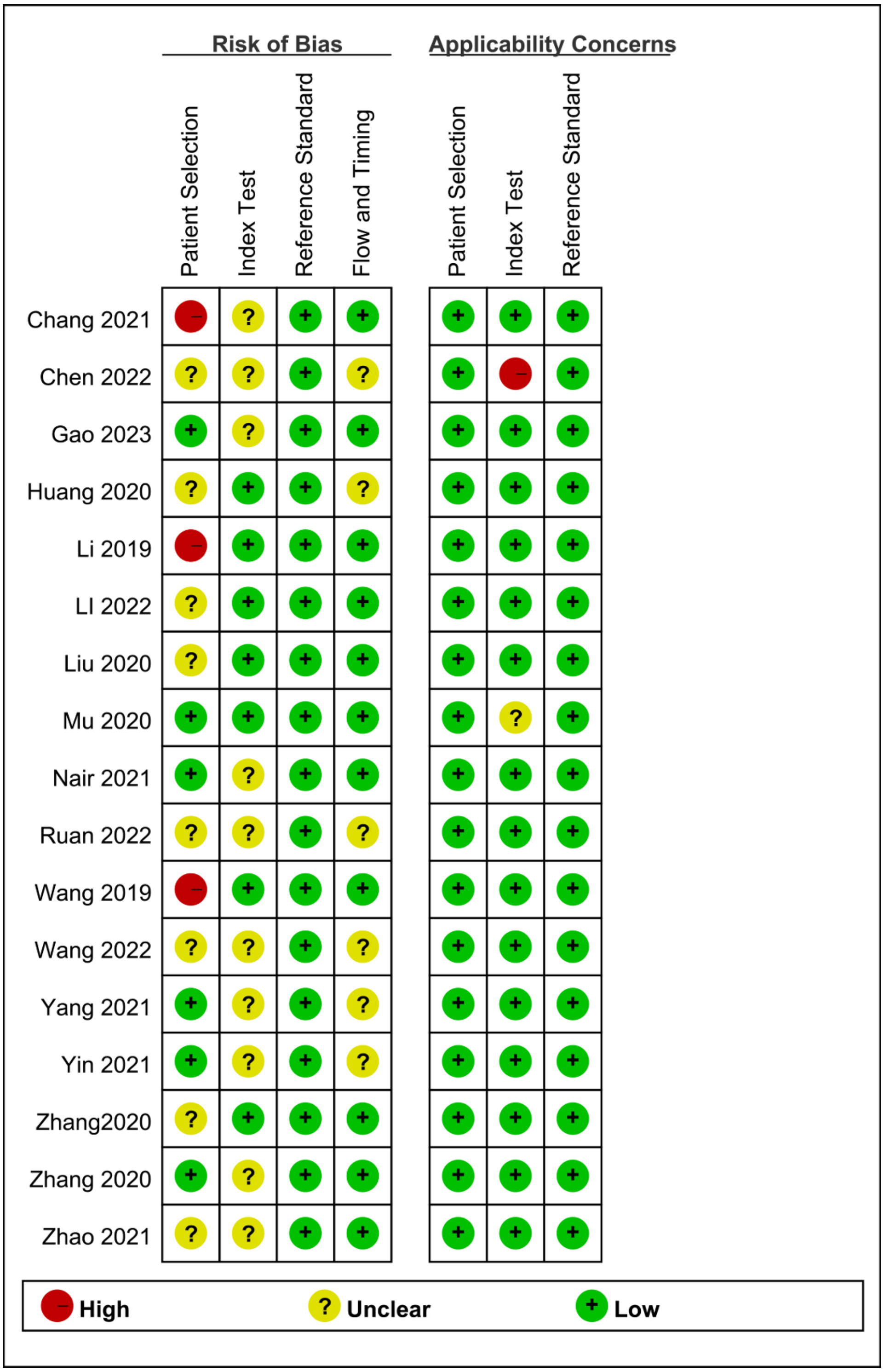

Figure S3 Summary of the risk of bias and applicability assessment: authors' judgement for each domain of each included study was reviewed. The proportion of included studies that indicated low, unclear, or high risk and applicability concerns are shown in green, yellow, and red, respectively

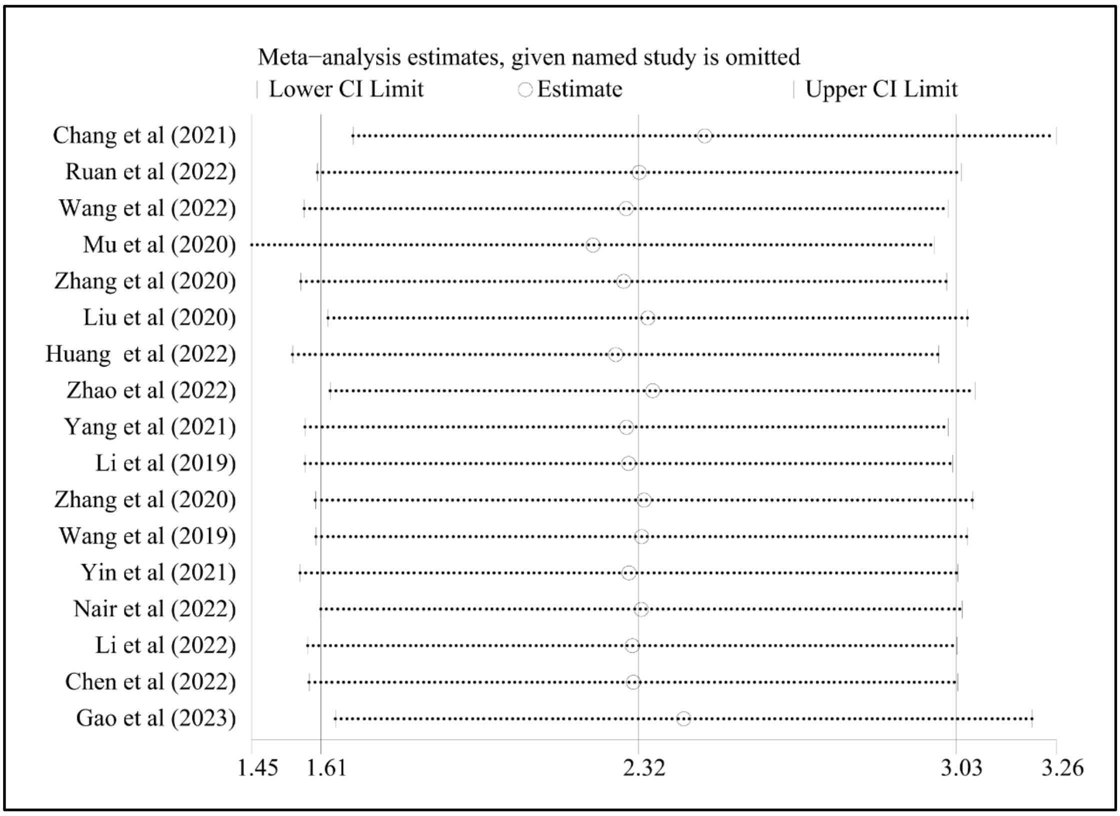

Figure S4 Sensitivity analysis of studies
